# Combination of GP88 Expression in Tumor Cells and Tumor-Infiltrating Immune Cells Is an Independent Prognostic Factor for Bladder Cancer Patients

**DOI:** 10.3390/cells10071796

**Published:** 2021-07-15

**Authors:** Markus Eckstein, Verena Lieb, Rudolf Jung, Danijel Sikic, Katrin Weigelt, Robert Stöhr, Carol Geppert, Veronika Weyerer, Simone Bertz, Ginette Serrero, Binbin Yue, Arndt Hartmann, Bernd Wullich, Helge Taubert, Sven Wach

**Affiliations:** 1 Institute of Pathology, University Hospital Erlangen, FAU Erlangen-Nürnberg, 91054 Erlangen, Germany; markus.eckstein@uk-erlangen.de (M.E.); Rudolf.Jung@uk-erlangen.de (R.J.); robert.stoehr@uk-erlangen.de (R.S.); carol.geppert@uk-erlangen.de (C.G.); veronika.weyerer@uk-erlangen.de (V.W.); simone.bertz@uk-erlangen.de (S.B.); Arndt.Hartmann@uk-erlangen.de (A.H.); 2 Bridge Consortium, 68135 Mannheim, Germany; danijel.sikic@uk-erlangen.de (D.S.); Bernd.Wullich@uk-erlangen.de (B.W.); sven.wach@uk-erlangen.de (S.W.); 3 Comprehensive Cancer Center Erlangen-EMN (CCC ER-EMN), 91054 Erlangen, Germany; Verena.Lieb@uk-erlangen.de (V.L.); Katrin.Weigelt@uk-erlangen.de (K.W.); 4 Department of Urology and Pediatric Urology, University Hospital Erlangen, FAU Erlangen-Nürnberg, 91054 Erlangen, Germany; 5 A&G Pharmaceutical Inc., Columbia, MD 21045, USA; gserrero@agpharma.com (G.S.); byue@agpharma.com (B.Y.); 6 Program in Oncology, University of Maryland Greenebaum Comprehensive Cancer Center, Baltimore, MD 21201, USA

**Keywords:** GP88, bladder cancer, prognosis, tumor cells, immune cells, lymph node stage

## Abstract

Urothelial bladder cancer (BCa) is the ninth most commonly diagnosed cancer worldwide and accounts for approximately 3% of global cancer diagnoses. We are interested in prognostic markers that may characterize tumor cells (TCs) and immune cells (ICs) and their relationship in BCa. A potential candidate marker that meets these criteria is progranulin (GP88), which is expressed separately in TCs and ICs. We analyzed GP88 expression by immunohistochemistry (IHC) in 196 muscle-invasive BCa samples using a tissue microarray. The immunoreactive score for GP88 staining in TCs and the percentage of GP88-positive ICs was determined. An easy cutoff for the staining status of TCs (positive vs. negative) and ICs (0% vs. >0%) and, more generally, negative vs. positive GP88 staining could be applied. We detected 93 patients (47.4%) and 92 patients (46.9%) with GP88-positive TCs or ICs, respectively. The IHC results were correlated with clinicopathological and survival data. Positive GP88 staining in TCs appeared to be an independent poor prognostic factor for disease-specific survival (DSS) (RR (relative risk) = 1.74; *p* = 0.009) and recurrence-free survival (RFS) (RR = 1.92; *p* = 0.002). In contrast, negative GP88 staining in ICs was an independent negative predictor for overall survival (OS) (RR = 2.18; *p* < 0.001), DSS (RR = 2.84; *p* < 0.001) and RFS (RR = 2.91; *p* < 0.001) in multivariate Cox’s regression analysis. When combining GP88 staining in TCs and ICs, a specific combination of GP88-positive TCs and GP88-negative ICs was associated with a 2.54-fold increased risk of death, a 4.21-fold increased risk of disease-specific death and a 4.81-fold increased risk of recurrence compared to GP88-negative TCs and GP88-positive ICs. In summary, GP88 positivity in TCs is a negative prognostic factor for DSS and RFS. In addition, GP88 positivity can mark ICs that are associated with a good prognosis (OS, DSS and RFS). The combination of GP88 staining in TCs and ICs appears to be a significant independent prognostic biomarker in muscle-invasive BCa.

## 1. Introduction

Urothelial bladder cancer (BCa) accounts for approximately 3% of global cancer diagnoses and 2.1% of cancer-related deaths worldwide [1]. Approximately 75% of BCa cases are non muscle-invasive BCa, and 25% of BCa cases are muscle-invasive BCa [2]. Currently, muscle-invasive BCa is treated by radical cystectomy with bilateral lymphadenectomy in combination with platinum-based perioperative chemotherapy in patients with extravesical tumors and/or lymph node metastasis [3,4].

In addition to tumor cells (TCs), the tumor immune microenvironment is associated with prognosis [5,6,7,8,9,10,11]. In particular, the presence of tumor infiltrating immune cells (ICs), such as leukocytes, including lymphocytes identified by their protein or gene expression profile, is associated with improved five-year overall survival (OS) or disease-specific survival (DSS) [8,10,11].

We were interested in potential biomarkers for bladder cancer that are expressed in TCs as well as in IC cells to gain more insight into the interaction of tumor cells and the tumor microenvironment. A well-recognized marker expressed in TCs and ICs in bladder cancer is PD-L1, which has already been utilized for therapy stratification of checkpoint inhibitor therapies [12,13,14]. Recently, we found that the expression of the immune cell marker chemokine CC motif ligand 2 (CCL2) was differentially associated with prognosis, depending on whether it was expressed in tumor cells (poor prognosis) or in immune cells (good prognosis) of bladder cancer patients [15]. Another candidate could be progranulin (GP88; synonymous: granulin–epithelin precursor; proepithelin; PC cell-derived growth factor; acrogranin) as it is expressed in bladder cancer cells [16] and is expected to be expressed in immune cells. GP88 protein expression has been shown in normal gastric mucosa in both epithelial and infiltrating immune cells (granulocytes, lymphocytes) [17]. One of the first articles about granulins described their presence in leukocytes [18], and another article described GP88 as a macrophage-derived factor [19]. However, none of the studies reported its presence, particularly in immune cells, in the context of tumors.

GP88, named after its molecular weight at ~88 kDa on Western immunoblots due to glycosylation, is physiologically a growth factor that regulates cell proliferation and wound repair, but pathologically it is also a component of the tumorigenesis machinery for different cancers [20,21,22,23]. Increased GP88 expression has been reported in breast cancer, brain tumors, non-small cell lung cancer, ovarian cancer, renal carcinoma, hepatocellular carcinoma, prostate cancer, colorectal cancer and hematological cancers [22,24,25,26,27,28,29,30]. An association between increased GP88 protein expression and a poor prognosis has been reported for breast cancer, ovarian cancer, non-small cell lung carcinoma, lymphomas, esophageal cancer, prostate cancer, and colorectal cancer [25,26,28,29,30,31,32]. GP88 expression is also correlated with resistance to anti-estrogen therapy in breast cancer patients [25,33] and platinum-based chemotherapy in non-small cell lung cancer patients [34].

In bladder cancer, in vitro studies demonstrated that GP88 stimulated the migration and invasion of bladder cancer cells [35]. Consistent with this finding, downregulation of GP88 in bladder cancer cells reduced their ability to proliferate in the absence of serum and inhibited migration, invasion and wound healing [36]. GP88-depleted urothelial cancer cells exhibited markedly reduced in vivo tumor growth in orthotopic and subcutaneous xenograft tumor models [16]. Progranulin depletion also sensitized urothelial cancer cells to cisplatin treatment [16,37]. Increased GP88 levels were detected in the urine of bladder cancer patients compared to healthy controls [36,38,39]. GP88 RNA expression was significantly upregulated in invasive bladder cancer tissues compared with normal urothelium, and elevated RNA expression was associated with a shorter OS [36]. Two immunohistochemical studies characterized GP88 protein expression in BCa [16,36]. Immunohistochemical staining in a small number of samples revealed that GP88 protein expression was significantly increased in invasive bladder tumors compared with normal bladder tissues [36]. Another study on a tissue microarray with 69 patients with BCa confirmed that GP88 protein expression was increased in tumor tissue compared with non-tumor bladder tissue [16]. In addition, GP88 was also overexpressed in metastatic bladder tissues, indicating that GP88 expression levels might be associated with bladder cancer metastasis. However, upregulation of GP88 was not significantly different between T1 and T2-4 urothelial carcinoma tissues [16]. Altogether, a statistical analysis of the association of GP88 protein expression with prognosis has not yet been reported in BCa.

The aim of this study was to examine GP88 protein expression in TCs and in ICs of muscle-invasive BCa, and to analyze the association of GP88 expression with clinicopathological parameters and prognosis.

## 2. Materials and Methods

### 2.1. Patients and Tumor Material

Tissue microarrays (TMAs) with formalin-fixed and paraffin-embedded tumor samples from 196 muscle-invasive BCa patients from the University Hospital Erlangen were investigated in this study. The TMA was prepared as follows: hematoxylin and eosin-stained slides were scanned (Panoramic P250, 3DHistech, Budapest, Hungary) and annotated using a TMA annotation tool (Caseviewer v2). Four cores (diameter 1 mm; two cores from the invasion margin, two cores from the tumor center) were taken utilizing an automated tissue microarrayer (TMA Grandmaster, 3DHistech, Budapest, Hungary), as described previously [13,40]. The research on human subjects was performed in compliance with the Helsinki Declaration. All patients provided written informed consent. The study is based on the approval of the Ethics Commission of the University Hospital Erlangen (No. 3755 and No. 329_16B). Tumor histology was reviewed by two uropathologists (AH, ME). An overview of the clinicopathological parameters of the patients included in this study is provided in Table 1.

### 2.2. Immunohistochemistry

For the study of GP88 protein expression, a manual IHC protocol was applied as previously described [29]. Briefly, after heat pretreatment at 120 °C for 5 min with TE–buffer pH 9 and peroxidase blocking (Dako, Hamburg, Germany), a primary antibody against GP88 (monoclonal mouse anti-GP88/PGRN antibody clone 6B3, Cat. No. AG10009; Precision Antibody, A&G Pharmaceutical, Columbia, MD, USA) was applied for 30 min. The stained specimens were viewed at objective magnifications of 10× and 20×. The slides were counterstained for 1 min with hematoxylin (Merck, Darmstadt, Germany). Between all of the steps, the slides were washed with buffer from Dako, and all of the incubation steps were performed at room temperature. For the study of the other proteins, staining was performed on a fully automated Ventana Benchmark Ultra autostainer (Ventana, Tucson, AZ, USA). IHC staining was performed with the following antibodies: CK5 (monoclonal mouse IgG, clone XM26; dilution 1:50; Diagnostic BioSystems, Pleasanton, CA, USA), CK20 (monoclonal mouse IgG, clone Ks 20.8; dilution 1:50; Dako, Hamburg, Germany), CD3 (monoclonal mouse IgG, clone F7.2.38; dilution 1:50; Thermo Fisher Scientific, Darmstadt, Germany), CD8 (mouse monoclonal IgG, clone C8/144B; dilution 1:50; Thermo Fisher Scientific), CD68 (mouse monoclonal IgG, clone PG-M1; dilution 1:60; Thermo Fisher Scientific), CD163 (mouse monoclonal IgG, clone 10D6; dilution 1:500; Novocastra/Leica, Wetzlar, Germany), PD-1 (NAT105, mouse monoclonal IgG; Ventana) and PD-L1 (SP263 assay, Ventana, Vreden, Germany). PD-L1+ immune cells (ICs) and tumor cells (TCs) were scored by two pathologists (AH, ME) according to the distributor’s PD-L1 scoring algorithm, as previously described [13]. Briefly, sections were deparaffinized, and antigens were retrieved by heating the sections in a pH 8.4 Tris/borate/EDTA solution (Ventana). Endogenous peroxidase was blocked with 1% H_2_O_2_. Visualization of a bound antibody was performed using the ultraVIEW TM DAB system (Ventana). All sections were counterstained with hematoxylin II/Mayer’s hematoxylin (Ventana).

Stained specimens were viewed by a certified uropathologist (M.E.) at magnifications of ×100 and ×200. Negative control slides without the addition of primary antibody were included for each staining experiment. From each sample, two cores from the center and two cores from the invasive front were analyzed. Afterwards, the staining average of both cores was determined, given that we did not observe significant differences between the two locations.

GP88 expression was detected in TCs (immunoreactive score; IRS) and characterized as positive (IRS > 0) or negative (IRS = 0) and in ICs (average of GP88 stained ICs in the invasion front and the tumor center) as the percentage of GP88-positive ICs out of all ICs. No relevant differences in staining intensities were noted. PD-L1 staining was determined as the percentage of stained TCs or ICs. Tumor-infiltrating lymphocytes were assessed on hematoxylin and eosin (H&E)-stained tissue sections as described previously, refs. [8,41] of the Figure S1 therein. For the survival analysis, patients were grouped as GP88 positive vs. negative in TCs and 0% GP88 cells vs. >0% GP88-positive ICs. Slides were scanned with a P250 slide scanner (3DHistech, Budapest, Hungary) and analyzed using CaseViewer 2.0 (3DHistech). Photos were obtained with a Leica DM 4000B microscope with a 20× HC PL Fluotar objective (Leica, Wetzlar, Germany) and a Jenoptik Gryphax Arktur camera (Jenoptik AG, Jena, Germany).

### 2.3. Immune Cell Quantification via Definiens Developer Software

CD3+, CD8+, and CD68+ ICs were quantified (counts per mm^2^) and log2-transformed for further analysis with Definiens Developer Software, as described previously [8].

### 2.4. Molecular Subtyping via NanoString Technology

RNA was isolated and purified as described previously [8]. The mRNA expression was determined via the nCounter^®^ MAX/FLEX system (NanoString Technologies^®^, Seattle, WA, USA) to determine luminal and basal phenotypes via a customized 21 gene containing nCounter^®^ PlexSet™ (NanoString Technologies^®^) according to the MD Anderson Cancer Center (MDA) subtyping approach [42,43], as previously described in detail by our group [8,10,44]. Gene counts were normalized using two reference genes (SDHA, HPRT1) and log2-transformed for further analysis with nSolver 4.0 software.

### 2.5. Statistical Analyses

The associations between the IHC and clinicopathological data were calculated using Spearman’s correlation test, the chi-squared test, or the Mann–Whitney test. The associations of GP88 expression with OS, DSS, and RFS were determined in univariate analyses (Kaplan–Meier analysis and Cox’s regression hazard models) and multivariate Cox’s regression analyses. Multivariate Cox’s regression analyses were adjusted for parameters that were significantly associated with prognosis in univariate Cox’s regression analysis, i.e., tumor stage, lymph node stage, and sex (Supplementary Table S3). A *p*-value of less than 0.05 was considered statistically significant. Statistical analyses were performed using the SPSS 21.0 software package (SPSS Inc., Chicago, IL, USA).

## 3. Results

### 3.1. GP88 Expression and Correlation with Clinicopathological Parameters and Expression of Selected Proteins

**Figure 1:**
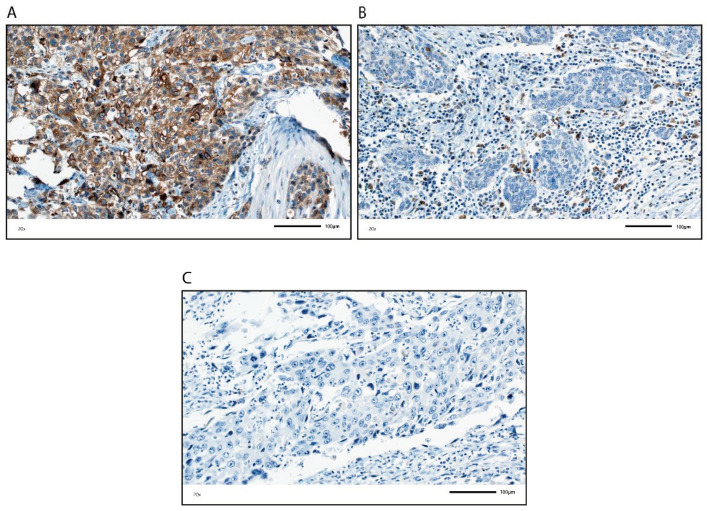
GP88 immunohistochemical staining in TCs and ICs. Upper row, (**A**) TCs, positive (ICs, negative); (**B**) positive ICs with >0% GP88 positivity (TCs, negative); lower row, (**C**) TCs and ICs, negative. All photomicrographs are at 20× objective magnification.

GP88 protein expression was assessed in a cohort of 196 muscle-invasive BCa patients using immunohistochemistry (IHC) (Figure 1 and Table 1). The clinicopathological data of the muscle invasive BCa patients are summarized in Table 1. GP88 protein expression was analyzed in TCs and in ICs individually. GP88 expression in TCs was scored based on an immunoreactive score (IRS) and classified as negative (IRS = 0) or positive (IRS > 0). For ICs, we determined the percentage of GP88-positive ICs in the tumor cell area and classified the ICs as negative (0% stained) or positive (>0% stained). We detected 103 patients (52.6%) and 104 patients (53.1%) with no GP88-stained TCs or ICs, and 93 patients (47.4%) and 92 patients (46.9%) with GP88-positive TCs or ICs, respectively (Supplementary Table S1). GP88 protein expression detected by IHC is shown in Figure 1.

Next, we analyzed whether GP88 staining was associated with clinicopathological and molecular parameters based on correlation tests.

In TCs, there was no association of GP88 staining with sex, age, tumor grade, or PD-L1 expression in TCs. A significant positive association was detected with tumor stage (r_s_ = 0.192; *p* = 0.007), lymph node stage (r_s_ = 0.276; *p* < 0.001), adjuvant chemotherapy (r_s_ = 0.164; *p* = 0.021), and molecular subtype (r_s_ = 0.159; *p* = 0.027).

A significant negative correlation was observed for GP88 TC staining and IC staining for GP88 (r_s_ = −0.369; *p* < 0.001), CD3 (r_s_ = −0.234; *p* = 0.001), CD8 (r_s_ = −0.200; *p* = 0.005), CCL2 on ICs (r_s_ = −0.181; *p* =0.020), PD1 on ICs (r_s_ = −0.216; *p* = 0.002), PD-L1 on ICs (r_s_ = −0.248; *p* < 0.001), percentage of stromal tumor infiltrating lymphocytes (TILs; r_s_ = −0.216; *p* = 0.002), overall survival status (r_s_ = −0.249; *p* < 0.001), disease-specific survival status (DSS: r_s_ = −0.343; *p* < 0.001), recurrence-free survival time (r_s_ = −0.141; *p* = 0.049), and recurrence-free survival status (RFS: r_s_ = −0.384; *p* < 0.001; Supplementary Table S2).

There was no association of GP88-positive IC with sex, age, or tumor stage. A significant positive association was found for GP88-positive IC and survival time (r_s_ = 0.321; *p* < 0.001), overall survival status (r_s_ = 0.273; *p* <0.001), disease-specific survival status (DSS) (r_s_ = 0.483; *p* < 0.001), recurrence-free survival time (r_s_ = 0.356; *p* < 0.001), recurrence-free survival status (RFS) (r_s_ = 0.495; *p* < 0.001), CK5 (r_s_ = 0.250; *p* < 0.001), percentage of stromal TILs (r_s_ = 0.424; *p* < 0.001), CD3 (r_s_ = 0.313; *p* < 0.001), CD8 (r_s_ = 0.299; *p* < 0.001), CD68 (r_s_ = 0.395; *p* < 0.001), CD163 (r_s_ = 0.253; *p* < 0.001), CCL2 on ICs (r_s_ = 0.349; *p* < 0.001), PD1 on ICs (r_s_ = 0.395; *p* < 0.001), PD-L1 expression on ICs (r_s_ = 0.437; *p* < 0.001), and PD-L1 expression on TCs (r_s_ = 0.150; *p* = 0.036).

A negative correlation for the GP88-positive IC was detected with tumor grade (r_s_ = −0.171; *p* = 0.017), lymph node stage (r_s_ = −0.196; *p* = 0.006), CK20 (r_s_ = −0.189; *p* = 0.008), adjuvant chemotherapy (r_s_ = −0.189; *p* = 0.008), and molecular subtype (r_s_ = −0.349; *p* < 0.001; Supplementary Table S2).

### 3.2. Association of GP88 Protein Expression in TCs and Survival

**Figure 2:**
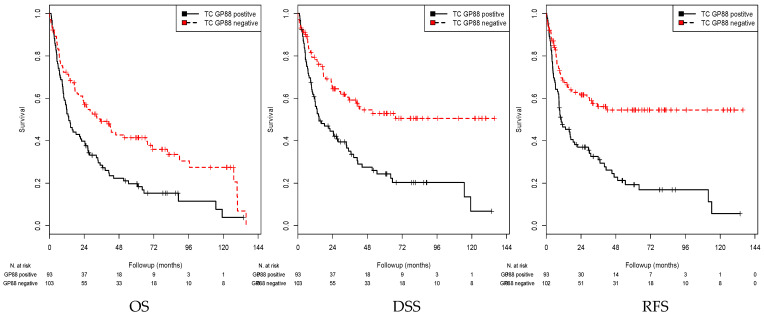
Kaplan–Meier analysis: Association between GP88 expression in TCs and prognosis. Positive GP88 expression in TCs was associated with a shorter mean OS (*p* = 0.001), mean DSS (*p* < 0.001), and mean RFS (*p* < 0.001) compared with negative GP88 expression.

GP88 staining in the TCs was considered positive (IRS > 0) or negative (IRS = 0). A significant association between positive GP88 staining and a shorter mean OS (*p* = 0.001), mean DSS (*p* < 0.001), and mean RFS (*p* < 0.001) was detected in the Kaplan–Meier analysis (log rank test) (Table 2 and Figure 2). When comparing patients with GP88-positive TCs with those with GP88-negative TCs, the mean OS was 32.5 months vs. 57.1 months, the mean DSS was 39.6 vs. 78.4 months, and the mean RFS was 33.5 vs. 79.1 months, respectively.

In univariate Cox’s regression analysis, GP88 positivity was associated with a 1.77-fold increased risk of death (*p* = 0.001), a 2.69-fold increased risk of disease-specific death (*p* < 0.001), and a 2.39-fold increased risk of recurrence (*p* < 0.001; Table 3).

The multivariate analysis was adjusted for the clinicopathological parameters that appeared to be significantly associated with prognosis in univariate Cox’s regression analysis, i.e., tumor stage, lymph node stage and sex (Supplementary Table S3). In multivariate Cox’s regression analysis, GP88 positivity in TCs appeared to be an independent poor prognostic factor for DSS (RR = 1.74; *p* = 0.009) and RFS (RR = 1.92; *p* = 0.002) and was identified as a trend for OS (RR = 1.41; *p* = 0.051; Table 3).

Next, we analyzed the association of GP88 expression in TC with prognosis (OS, DSS, RFS) in different patient subgroups (Table 2 and Table 3).

In these subgroup analyses, the negative prognostic value of GP88 staining on TCs was individually confirmed among patients with tumor stage 3+4, those with nodal stage N0, patients not treated with chemotherapy (CT−), as well as those with basal and especially luminal tumors in univariate analyses (Table 2 and Table 3). Multivariate Cox’s regression analysis further revealed an increased risk for patients with nodal stage N0, patients not treated with chemotherapy (CT−) and luminal tumors for OS, DSS and RFS (Table 3). In addition, an increased risk for recurrence was observed for tumor stage 3+4 patients (Table 3).

### 3.3. Association of GP88 Protein Expression in ICs and Survival

**Figure 3:**
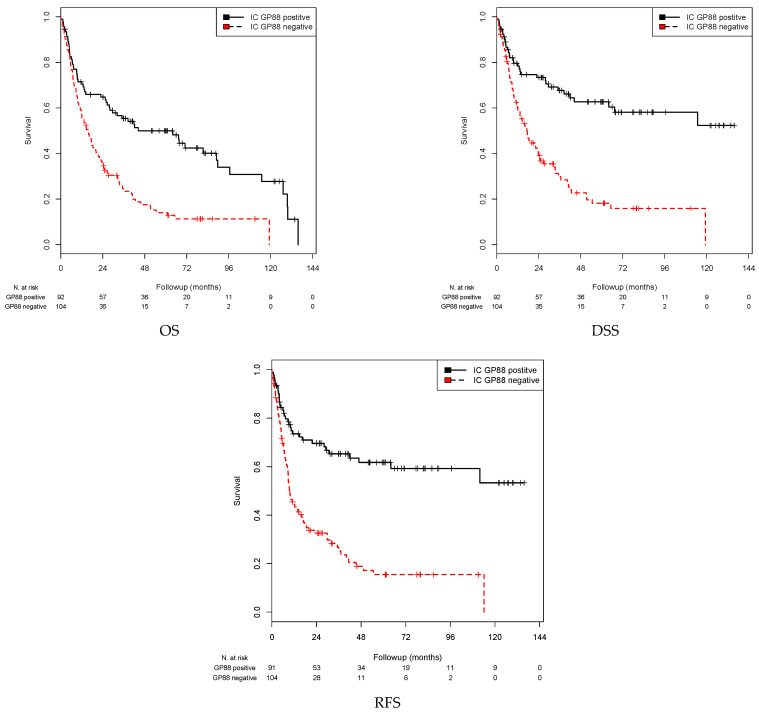
Kaplan–Meier analysis: association between GP88 expression in ICs and prognosis. Negative GP88 expression in ICs was associated with a shorter mean OS (*p* < 0.001), mean DSS (*p* < 0.001), and mean RFS (*p* < 0.001) compared with positive GP88 expression.

For statistical survival analysis, patients were separated into GP88-negative ICs (0%) and GP88-positive ICs (>0%). A significant association of GP88-positive ICs with OS, DSS and RFS (all *p* < 0.001) was observed by Kaplan-Meier analysis (Table 4 and Figure 3). When comparing the patients with GP88-positive ICs with those with GP88-negative ICs, the mean OS was 62.9 months vs. 29.4 months, the mean DSS was 86.3 months vs. 34.5 months, and the mean RFS was 85.5 vs. 29.4 months, respectively (Table 4).

In the univariate Cox’s regression analysis, GP88 negativity in ICs was associated with a 2.27-fold increased risk of death, a 3.04-fold increased risk of disease-specific death, and a 3.18-fold increased risk of recurrence (all *p* < 0.001; Table 5). Multivariate Cox’s regression analysis (adjusted for tumor stage, lymph node stage, and sex) showed that GP88-negative stained ICs were an independent predictor of shorter OS (RR = 2.18; *p* < 0.001), DSS (RR = 2.84; *p* < 0.001) and RFS (RR = 2.91; *p* < 0.001; Table 5). Altogether, in contrast to TCs, GP88 positivity in ICs appears to be a favorable prognostic factor.

Next, the association of GP88 protein expression in ICs and survival was stratified by clinicopathological and molecular parameters in subgroup analyses.

In these subgroup analyses, the negative prognostic value of negative GP88 staining on ICs was individually confirmed among patients with tumor stage 3+4, those with nodal stage N0, patients not treated with chemotherapy (CT−), as well as those with basal and luminal tumors (Table 4 and Table 5). Multivariate Cox’s regression analysis further revealed an increased risk for patients with tumor stage 3+4, nodal stage N0, patients treated or not treated with chemotherapy (CT−), and with basal or luminal tumors for OS, DSS and RFS (Table 5).

### 3.4. Association of Combined GP88 Staining in TCs and ICs with Prognosis

**Figure 4:**
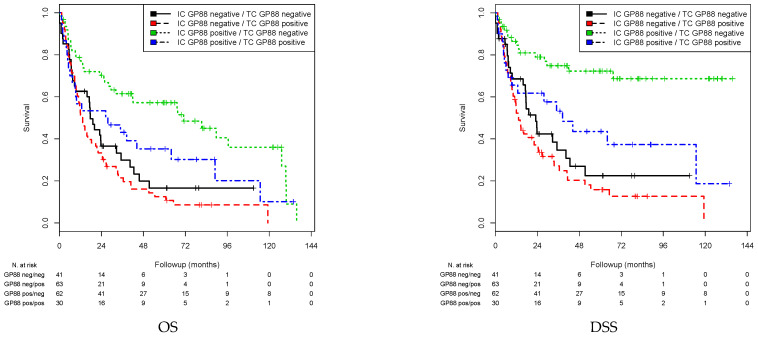

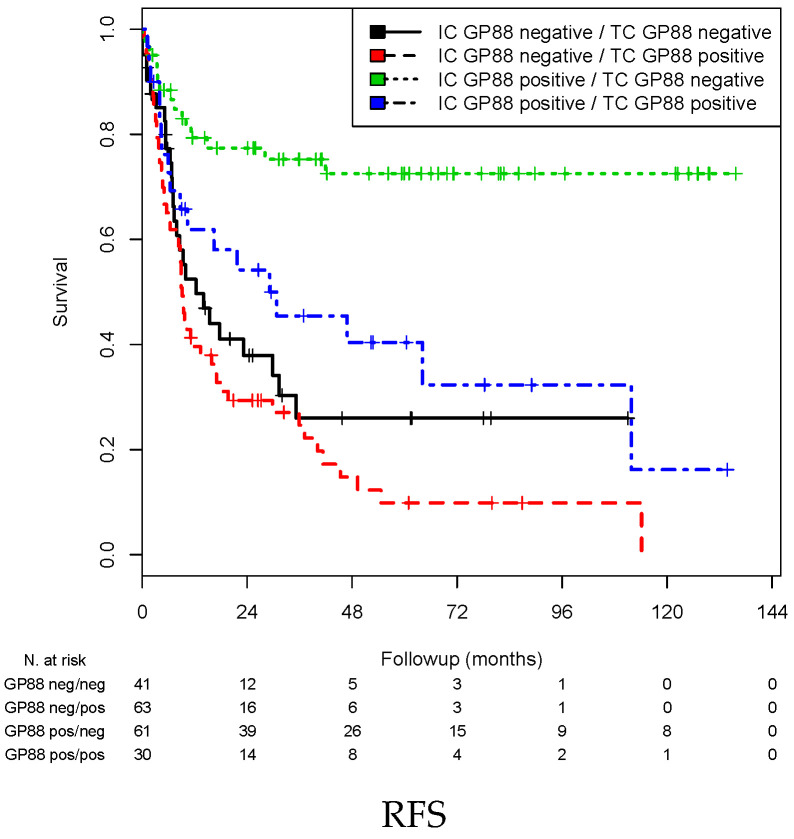
Kaplan–Meier analysis: association between combined GP88 expression in TCs + ICs and prognosis.

Finally, we investigated how a combination of different GP88 staining in the TCs and ICs was associated with prognosis to define groups with worse and better prognosis. We separated the patients into four groups for GP88 staining: (i) TC negative and IC positive; (ii) TC negative and IC negative; (iii) TC positive and IC positive; and (iv) TC positive and IC negative. Consistent with the findings in the single analyses, group i (TC negative, IC positive) showed the best prognosis (reference group), and group iv (TC positive and IC negative) exhibited the worst prognosis. In Kaplan–Meier analysis, patients in group (i) survived 70.88 months, and those in group iv survived 26.45 months (*p* < 0.001). Patients in group (ii) (33.51 months) and group (iii) (46.20 months) exhibited survival rates in between these values (Table 6; Figure 4). Comparably, in group (i), DSS (99.65 months) and RFS (101.53 months) were the best, and DSS (30.80 months) and RFS (24.46 months) were the worst in group (iv) (*p* < 0.001; Table 6; Figure 4).

In univariate Cox’s regression analysis, patients in group (iv) showed a 3.00-fold increased risk for death (*p* < 0.001), a 5.11-fold increased risk for disease-specific death and a 5.75-fold increased risk for recurrence compared to patients in group i (Table 7).

In multivariate Cox’s regression analysis (adj. for sex, tumor stage, nodal stage) patients in the (iv) group possessed a 2.54-fold increased risk for death, a 4.21-fold increased risk for disease-specific death and a 4.81-fold increased risk for recurrence compared to patients in group (i) (all *p* < 0.001; Table 7). Patients in group (ii) (TC and IC negative) also showed a significantly increased risk for death (RR = 2.67), disease-specific death (RR = 4.21) and recurrence (RR = 4.26; all *p* < 0.001) compared to the reference group (i). Interestingly, patients in group (iii) did not differ in their risk for death but in their risk for disease-specific death (RR = 2.42; *p* < 0.014) and risk for recurrence (RR = 2.67; *p* = 0.006) from the reference group. Altogether, GP88 staining of ICs had a more profound effect on prognosis than TC staining. However, a combined evaluation of staining of TCs and ICs allowed further substratification of BCa patients for prognosis.

GP88-negative TC/GP88-positive IC patients served as the reference group (green line). Patients with GP88 positivity in TCs/GP88 negativity in ICs exhibited the worst prognosis (OS, DSS, RFS: all *p* < 0.001; red line). The second worst group included patients who were GP88 negative in both TCs and ICs, and the second best prognostic group included patients who were GP88 positive in TCs and ICs.

## 4. Discussion

We analyzed GP88 protein expression in TCs and in ICs in tumors from BCa patients by IHC and assessed its association with clinicopathological and survival data. Remarkably, GP88 staining was inversely correlated in the TCs and in the ICs. GP88 staining in TCs was positively correlated with tumor stage, lymph node stage, adjuvant chemotherapy and molecular subtype, and negatively correlated with immune cells and their markers as well as with OS, DSS and RFS.

In contrast, GP88 staining of ICs was positively correlated with the presence of immune cells and their markers, and with prognosis (OS, DSS, RFS). In addition, GP88-positive IC staining was negatively correlated with tumor grade, lymph node stage, adjuvant chemotherapy and molecular subtype. The correlation of GP88 positivity in ICs to different immune cell markers suggests that various immune cells may express GP88, but this issue awaits further clarification in future studies with staining for multiple proteins.

Previous reports of GP88 expression have exclusively concentrated on investigating GP88 expression in the tumor cells. GP88 expression has been shown to enhance proliferation and promote tumor growth in several cancer cell lines and cancers, e.g., breast, gastrointestinal, hepatic, lung, and genitourinary cancers [22,23,27]. In bladder cancer, in addition to extensive in vitro and in vivo model studies [16,35,36], tumor cells showed increased GP88 expression at the RNA and protein levels compared with normal tissue [16,36]. Furthermore, GP88 was overexpressed in metastatic bladder tissues, but Buraschi et al. could not identify significant differential upregulation of GP88 between T1 and T2-4 urothelial carcinoma tissues [16]. In this present study, we exclusively focused on muscle-invasive BCa with tumor stages ranging from pT2 to pT4. We found a correlation between increased GP88 protein expression in TCs and higher tumor stage.

We showed for the first time that GP88 expression in TCs is an independent negative predictor for DSS and RFS in muscle-invasive BCa patients. After patient stratification based on several clinicopathological and molecular parameters, multivariate analysis revealed that GP88 expression in TCs was an independent prognostic factor, i.e., for OS, DSS and RFS in the pT3+4 group, N0 group, CT−group and luminal subgroup. Thus, a more detailed characterization can be made of, for example, the pT3+4 group, which is generally considered to have a poor prognosis; specifically, patients with a poor prognosis (TC GP88 positive) and patients with a better prognosis (TC GP88 negative) can be distinguished. Of special interest is that GP88 expression has been correlated with resistance to platinum-based chemotherapy in non-small cell lung cancer patients, and progranulin depletion sensitizes urothelial cancer cells to cisplatin treatment [16,34,37]. However, until now, pretreatment gene signatures for the luminal muscle-invasive BCa subtypes that might predict chemosensitivity were not available [42]. GP88 positivity in TCs predicts poor outcomes in the absence of chemotherapy, but not for patients treated with chemotherapy, which could indicate a chemotherapy-induced cancellation of the adverse effect of GP88 expression on TCs.

In addition, we report for the first time that GP88 expression in ICs is an independent positive predictor for OS, DSS and RFS in muscle-invasive bladder cancer patients. After stratification of the patients for several clinicopathological and molecular parameters, we found in multivariate analysis that negative GP88 expression in ICs was an independent negative prognostic factor (OS, DSS and RFS) in the pT3+4 group, in the CT− and CT+, as well as basal and luminal subgroups. However, an association was also noted between negative GP88 expression in ICs and poor RFS in the pT2 group.

Why are GP88-positive ICs associated with better prognosis? Tumors can be considered as wounds that do not heal [45]. GP88 is a growth factor that has been described in wound-healing [46]. In a mouse wound healing model, He et al. observed that inflammatory cells infiltrating the wound, including neutrophils and macrophages, expressed progranulin mRNA from day one [46]. In addition, when GP88 was applied to a cutaneous wound, it increased the accumulation of immune cells (neutrophils, macrophages), blood vessels and fibroblasts in the wound.

It is known that GP88 can be produced by immune cells such as leukocytes [17,18,19,47]. The presence of different immune cells (TILs) has been associated with better prognosis and therapy response in muscle invasive bladder cancer [10,48,49]. For tumor cells, GP88 might function as an autocrine growth factor [25], while in the context of immune cells, its anti-inflammatory function might be predominant [50]. Moreover, there are several points at which GP88 can be active in the immune system with an anti-tumorigenic activity: (i) GP88 is involved in the activation of innate immunity by serving as a soluble cofactor for TLR9 e.g., in macrophages [51]. (ii) GP88 promotes the Th2 (T-helper 2 cells) response [52], and can induce expression of Th2-like cytokines, IL-4 and IL-5 [53]. Components of the Th2-mediated immune response, such as IL-4 and eosinophils, can decrease tumor growth and initiate anti-tumor activity [54]. (iii) Binding of GP88 to TNFR2 inhibits binding of TNFα [55]. TNFα is known for its immunosuppressive activity by intrinsic negative effect on conventional T cells activation and by boosting suppressive cells, as myeloid-derived suppressor cells (MDSC) or regulatory T cells (Tregs) [56].

Altogether, our findings suggest that GP88 expression could mark a population of ICs that may be anti-tumorigenic in muscle-invasive BCa patients.

Finally, we focused on the combination of GP88 staining in TCs and ICs and their association with prognosis to define patient groups with worse and better prognoses. GP88 staining in TCs and ICs was negatively correlated. As expected from the single GP88 staining analyses, the group with TC-negative and IC-positive GP88 staining showed the best prognosis. In contrast, the TC-positive/IC-negative group presented the worst prognosis, whereas the other two groups were prognostically in between for OS, DSS and RFS. The combination of GP88 staining of TCs and ICs showed an additive effect for risk evaluation compared to the single analyses in either TCs or ICs. Remarkably, for the TC-positive/IC-negative group, a greater than 2.5-fold risk for death and a greater than 4-fold increased risk for either disease-specific death or recurrence could be calculated. This finding implies that GP88 evaluated in both TCs and ICs could be a relevant prognostic marker for muscle-invasive BCa cancer patients. However, targeting GP88 in tumor therapy by siRNA or GP88 monoclonal antibodies, as recently shown in a triple-negative breast cancer tumor xenograft model [57], might be restricted to TCs and should possibly spare ICs in BCa.

Our study has some limitations. This was a retrospective study. After stratification, the patient sub-groups were rather small. Therefore, our results must be evaluated in a larger prospective study. However, altogether, the number of patients in this study allowed for the reasonable multivariate analysis of the association of one parameter, namely GP88 staining, with prognosis in muscle-invasive BCa patients. The patients were not treated with checkpoint inhibitor immunotherapy, given the retrospective nature of the study. However, GP88 expression (as PD-L1 expression) in TCs was associated with poor prognosis, whereas GP88 expression (as PD-L1 protein expression) in ICs was associated with a better prognosis. GP88 expression in TCs can be associated with chemotherapy resistance in different cancers/cancer cells. Thus, GP88 expression may have some predictive potential for future chemo- and immunotherapies, in addition to its prognostic value.

However, further characterization of the type of ICs expressing GP88 is necessary.

## 5. Conclusions

Altogether, GP88 positivity in TCs is an independent negative prognostic factor for DSS and RFS. In contrast, GP88 positivity in ICs is an independent positive prognostic marker for OS, DSS and RFS. By combining GP88 staining results in the TCs and in the ICs, in a group with the worst prognosis (GP88 negative ICs/GP88 positive TCs) and a group with a superior prognosis (GP88 positive ICs/GP88 negative TCs) could be identified. Given that GP88 staining in TCs can be predictive for chemotherapy and GP88 staining in ICs marks a group of ICs associated with a good prognosis, GP88 staining in TCs and ICs may have predictive potential for chemo-/immunotherapies for muscle-invasive BCa patients.

## Figures and Tables

**Table 1:** Clinicopathological data and survival parameters of the BCa patients.

Clinicopathological and Survival Parameters	Patients (Percentage)
Total	196
**Age (years)**	
range	37–91
mean	69.5
median	71.0
**Sex**	
Female	55 (28.1)
Male	141 (71.9)
**Morphology**	
NOS	105 (53.6)
Squamous	42 (21.4)
Sarcomatoid	10 (5.1)
MPUC	9 (4.6)
PUC	7 (3.6)
Pure neuroendocrine	10 (5.1)
Other rare subtypes	13 (6.6)
**Tumor Stage**	
pT2	53 (27.0)
pT3	97 (49.5)
pT4	46 (23.5)
**Tumor Grade 1973**	
G2	6 (3.1)
G3	190 (96.9)
**Tumor Grade 2016**	
High grade	196 (100.0)
**Nodal Stage**	
pN0	124 (63.3)
pN1+2	57 (29.1)
pNX	15 (7.6)
**Adjuvant Chemotherapy (Ct)**	
Yes	50 (25.5)
No	146 (74.5)
**Stromal TILs**	
0	15 (7.7)
1–9%	72 (36.7)
10–50%	93 (47.5)
51–80%	14 (7.1)
>80%	2 (1.0)
**Molecular Subtypes**	
Basal	88 (44.9)
DN	11 (5.6)
Luminal	75 (38.3)
Luminal EMT-p53-like	21 (10.7)
Not specified	1 (0.5)
**Survival/observation Time (months)**	
Range (months)	0–135.7
Mean	33.3
Median	21.1
**Overall Survival Status**	
Dead	145 (74.0)
Alive	51 (26.0)
**Disease-specific Survival Status**	
Dead	110 (56.1)
Alive	86 (43.9)
**Recurrence-free Survival Time (months)**	
Range	0–135.7
Mean	29.8
Median	14.2
**Recurrence**	
Yes	113 (57.7)
No	83 (42.3)

Abbreviations: DN = double negative for basal and luminal markers; TILs = tumor-infiltrating lymphocytes; NOS = not otherwise specified; MPUC = micropapillary urothelial carcinoma; PUC = plasmacytoid urothelial carcinoma.

**Table 2:** Kaplan–Meier analysis: Association of GP88 staining in TCs with mean OS, mean DSS, or mean RFS.

Parameter	Kaplan-Meier Analysis							
GP88	N	OS		DSS		N	RFS	
positive vs. negative								
		**Months**	***p***	**Months**	***p***		**Months**	***p***
All Patients	196	32.5 vs. 57.1	**0.001**	39.6 vs. 78.4	**<0.001**	195	33.5 vs. 79.1	**<0.001**
Tumor Stage 2	53	47.9 vs. 81.9	0.070	66.0 vs. 93.3	n.s.	53	57.2 vs. 92.9	**0.049**
Tumor Stage 3+4	143	29.6 vs. 46.6	**0.015**	33.2 vs. 68.3	**0.001**	142	27.8 vs. 69.2	**<0.001**
Nodal Stage N0	124	42.9 vs. 68.1	**0.016**	53.6 vs. 92.3	**0.001**	123	45.9 vs. 94.2	**<0.001**
Nodal Stage N1/N2	57	27.2 vs. 22.3	n.s.	32.1 vs. 26.7	n.s.	57	25.3 vs. 24.2	n.s.
Nodal Stage NX	15	8.9 vs. 17.5	n.s.	8.9 vs. 17.5	n.s.	15	6.1 vs. 14.2	n.s.
CT−	146	30.0 vs. 58.5	**0.001**	38.3 vs. 82.1	**<0.001**	145	34.2 vs. 83.9	**<0.001**
CT+	50	40.2 vs. 49.6	n.s.	43.5 vs. 58.5	n.s.	50	32.7 vs. 56.1	n.s.
Basal	88	35.4 vs. 62.5	(0.051)	47.4 vs. 90.9	**0.005**	88	38.9 vs. 91.5	**0.002**
Luminal	75	32.5 vs. 66.2	**0.002**	38.6. vs. 71.4	**0.003**	74	34.2 vs. 72.4	**0.002**
Luminal EMT-p53-like	21	26.5 vs. 32.0	n.s.	28.7 vs. 48.6	n.s.	21	24.8. vs. 49.1	n.s.
DN	11	10.1 vs. 31.5	n.s.	10.1 vs. 37.3	n.s.	11	7.9 vs. 36.8	n.s.

Abbreviations: Basal = basal subtype; Luminal = luminal subtype; Luminal EMT-p53 = luminal EMT-p53 subtype; CT = adjuvant chemotherapy; n.s. = not significant.

**Table 3:** Univariate and multivariate Cox’s regression analysis: association of GP88 staining in TCs with mean OS, mean DSS, or mean RFS.

**Parameter**	**Univariate Cox’s Regression Analysis**							
**GP88**	**N**	**OS**		**DSS**		**N**	**RFS**	
positive vs. negative								
		**RR**	***p***	**RR**	***p***		**RR**	***p***
All Patients	196	1.77	**0.001**	2.69	**<0.001**	195	2.39	**<0.001**
Tumor Stage 2	53	n.s.	n.s.	n.s.	n.s.	53	n.s.	n.s.
Tumor Stage 3+4	143	1.59	**0.016**	2.09	**0.001**	142	2.16	**0.001**
Nodal Stage N0	124	1.72	**0.017**	2.37	**0.002**	123	2.58	**0.001**
Nodal Stage N1/N2	57	n.s.	n.s.	n.s.	n.s.	57	n.s.	n.s.
Nodal Stage NX	15	n.s.	n.s.	n.s.	n.s.	15	n.s.	n.s.
CT−	146	1.86	**0.001**	2.50	**<0.001**	145	2.59	**<0.001**
CT+	50	n.s.	n.s.	n.s.	n.s.	50	n.s.	n.s.
Basal	88	1.65	(0.053)	2.38	**0.007**	88	2.61	**0.002**
Luminal	75	2.52	**0.002**	2.55	**0.004**	74	2.73	**0.003**
Luminal EMT-p53-like	21	n.s.	n.s.	n.s.	n.s.	21	n.s.	n.s.
DN	11	n.s.	n.s.	n.s.	n.s.	11	n.s.	n.s.
	**Multivariate Cox’s Regression Analysis**							
**GP88**	**N**	**OS**		**DSS**		**N**	**RFS**	
positive vs. negative								
		RR	*p*	RR	*p*		RR	*p*
All Patients	196	1.41	(0.051)	1.74	**0.009**	195	1.92	**0.002**
Tumor Stage 2	53	n.s.	n.s.	n.s.	n.s.	53	n.s.	n.s.
Tumor Stage 3+4	143	n.s.	n.s.	1.62	(0.053)	142	1.69	**0.035**
Nodal Stage N0	124	1.67	**0.031**	2.31	**0.004**	123	2.49	**0.001**
Nodal Stage N1/N2	57	n.s.	n.s.	n.s.	n.s.	57	n.s.	n.s.
Nodal Stage NX	15	n.s.	n.s.	n.s.	n.s.	15	n.s.	n.s.
CT−	146	1.58	**0.027**	2.01	**0.005**	145	2.13	**0.002**
CT+	50	n.s.	n.s.	n.s.	n.s.	50	n.s.	n.s.
Basal	88	n.s.	n.s.	n.s.	n.s.	88	1.81	(0.091)
Luminal	75	2.22	**0.012**	2.18	**0.023**	74	2.66	**0.007**
Luminal EMT-p53-like	21	n.s.	n.s.	n.s.	n.s.	21	n.s.	n.s.
DN	11	n.s.	n.s.	n.s.	n.s.	11	n.s.	n.s.

Abbreviations: basal = basal subtype; luminal = luminal subtype; luminal EMT-p53 = luminal EMT-p53 subtype; CT = adjuvant chemotherapy; n.s. = not significant; significant *p*-values are marked in bold face.

**Table 4:** Kaplan–Meier analysis: association of GP88 staining in ICs with mean OS, mean DSS, or mean RFS.

Parameter	Kaplan-Meier Analysis							
GP88	N	OS		DSS		N	RFS	
positive vs. negative								
		**Months**	***p***	**Months**	***p***		**Months**	***p***
All Patients	196	62.9 vs. 29.4	**<0.001**	86.3 vs. 34.5	**<0.001**	195	85.5 vs. 29.4	**<0.001**
Tumor Stage 2	53	75.4 vs. 52.6	n.s.	99.2 vs. 59.9	(0.060)	53	99.2 vs. 54.1	**0.022**
Tumor Stage 3+4	143	57.7 v. 21.0	**0.030**	80.3 vs. 24.2	**<0.001**	142	78.9 vs. 19.5	**<0.001**
Nodal Stage N0	124	80.8 vs. 32.9	**0.001**	110.2 vs. 38.5	**<0.001**	123	109.6 vs. 34.6	**<0.001**
Nodal Stage N1/N2	57	21.4 vs. 28.4	n.s.	25.8 vs. 32.8	n.s.	57	23.1 vs. 26.1	n.s.
Nodal Stage NX	15	20.1 vs. 8.0	n.s.	20.1 vs. 8.0	n.s.	15	16.9 vs. 5.1	(0.081)
CT−	146	63.4 vs. 26.2	**<0.001**	89.2 vs. 31.5	**<0.001**	145	90.6 vs. 28.2	**<0.001**
CT+	50	59.0 vs. 36.2	**0.043**	70.7 vs. 38.4	**0.033**	50	61.7 vs. 31.3	(0.059)
Basal	88	64.7 vs. 23.4	**0.001**	94.3 vs. 26.2	**<0.001**	88	91.6 vs. 20.7	**<0.001**
Luminal	75	65.7 vs. 33.9	**0.006**	71.6 vs. 39.8	**0.008**	74	73.8 vs. 35.4	**0.006**
Luminal EMT-p53-like	21	33.4 vs. 28.6	n.s.	39.9 vs. 34.9	n.s.	21	40.9 vs. 30.2	n.s.
DN	11	52.2 vs, 13.5	n.s.	74.1 vs. 13.5	(0.072)	11	74.1 vs. 7.3	**0.044**

Abbreviations: basal = basal subtype; luminal = luminal subtype; luminal EMT-p53 = luminal EMT-p53 subtype; CT = adjuvant chemotherapy; n.s. = not significant.

**Table 5:** Univariate and multivariate Cox’s regression analysis: association of GP88 staining in ICs with mean OS, mean DSS, or mean RFS.

**Parameter**	**Univariate Cox’s Regression Analysis**							
**GP88**	**N**	**OS**		**DSS**		**N**	**RFS**	
positive vs. negative								
		**RR**	***p***	**RR**	***p***		**RR**	***p***
All Patients	196	2.27	**<0.001**	3.04	**<0.001**	195	3.18	**<0.001**
Tumor Stage 2	53	n.s.	n.s.	n.s.	n.s.	53	2.77	**0.028**
Tumor Stage 3+4	143	2.49	**<0.001**	3.20	**<0.001**	142	3.25	**<0.001**
Nodal Stage N0	124	3.34	**<0.001**	6.40	**<0.001**	123	6.58	**<0.001**
Nodal Stage N1/N2	57	n.s.	n.s.	n.s.	n.s.	57	n.s.	n.s.
Nodal Stage NX	15	n.s.	n.s.	n.s.	n.s.	15	n.s.	n.s.
CT−	146	2.39	**<0.001**	3.35	**<0.001**	145	3.62	**<0.001**
CT+	50	2.12	**0.048**	2.37	**0.037**	50	2.07	(0.064)
Basal	88	2.43	**0.001**	3.44	**<0.001**	88	3.72	**<0.001**
Luminal	75	2.33	**0.007**	2.41	**0.010**	74	2.57	**0.007**
Luminal EMT-p53-like	21	n.s.	n.s.	n.s.	n.s.	21	n.s.	n.s.
DN	11	n.s.	n.s.	n.s.	n.s.	11	6.71	(0.078)
	**Multivariate Cox’s Regression Analysis**							
**GP88**	**N**	**OS**		**DSS**		**N**	**RFS**	
positive vs. negative								
		**RR**	***p***	**RR**	***p***		**RR**	***p***
All Patients	196	2.18	**<0.001**	2.84	**<0.001**	195	2.91	**<0.001**
Tumor Stage 2	53	n.s.	n.s.	n.s.	n.s.	53	4.51	**0.004**
Tumor Stage 3+4	143	2.27	**<0.001**	2.88	**<0.001**	142	2.88	**<0.001**
Nodal Stage N0	124	3.83	**<0.001**	7.68	**<0.001**	123	7.21	**<0.001**
Nodal Stage N1/N2	57	n.s.	n.s.	n.s.	n.s.	57	n.s.	n.s.
Nodal Stage NX	15	n.s.	n.s.	n.s.	n.s.	15	n.s.	n.s.
CT−	146	2.36	**<0.001**	3.27	**<0.001**	145	3.56	**<0.001**
CT+	50	3.02	**0.001**	2.39	**0.008**	50	2.73	**0.020**
Basal	88	2.10	**0.013**	3.06	**0.002**	88	3.37	**0.001**
Luminal	75	2.71	**0.007**	2.72	**0.014**	74	2.62	**0.015**
Luminal EMT-p53-like	21	n.s.	n.s.	n.s.	n.s.	21	n.s.	n.s.
DN	11	n.s.	n.s.	n.s.	n.s.	11	n.s.	n.s.

Abbreviations: basal = basal subtype; luminal = luminal subtype; luminal EMT-p53 = luminal EMT-p53 subtype; CT = adjuvant chemotherapy; n.s. = not significant; significant *p*-values are marked in bold face.

**Table 6:** Kaplan–Meier analysis: association of combined GP88 staining in TCs and ICs with mean OS, mean DSS, or mean RFS.

Parameter	Kaplan-Meier Analysis							
GP88	N	OS		DSS		N	RFS	
		Months	*p*	Months	*p*		Months	*p*
TC negative/IC positive	62	70.88		99.65		61	101.53	
TC negative/IC negative	41	33.51		39.70		41	37.63	
TC positive/IC positive	30	46.20		59.12		30	53.16	
TC positive/IC negative	63	26.45	**<0.001**	30.80	**<0.001**	63	24.46	**<0.001**

**Table 7:** Univariate and multivariate Cox’s regression analysis: association of combined GP88 staining in TCs and ICs with mean OS, mean DSS, or mean RFS.

**Parameter**	**Univariate Cox’s Regression Analysis**							
**GP88**	**N**	**OS**		**DSS**		**N**	**RFS**	
		**RR**	***p***	**RR**	***p***		**RR**	***p***
TC negative/IC positive	62	reference		reference		61	reference	
TC negative/IC negative	41	2.39	**0.001**	3.84	**<0.001**	41	4.20	**<0.001**
TC positive/IC positive	30	1.75	**0.041**	2.83	**0.003**	30	3.13	**0.001**
TC positive/IC negative	63	3.00	**<0.001**	5.11	**<0.001**	63	5.75	**<0.001**
	**Multivariate Cox’s Regression Analysis**							
**GP88**	**N**	**OS**		**DSS**		**N**	**RFS**	
		**RR**	***p***	**RR**	***p***		**RR**	***p***
TC negative/IC positive	62	reference		reference		61	reference	
TC negative/IC negative	41	2.67	**<0.001**	4.21	**<0.001**	41	4.26	**<0.001**
TC positive/IC positive	30	n.s.	n.s.	2.42	**0.014**	30	2.67	**0.006**
TC positive/IC negative	63	2.54	**<0.001**	4.21	**<0.001**	63	4.81	**<0.001**

## Data Availability

All data are available in the manuscript and the Supplementary Materials. Detailed datasets used and analyzed during the current study are available from the corresponding author on reasonable request.

## Supplementary Materials

The following data are available online at https://www.mdpi.com/article/10.3390/cells10071796/s1, Table S1: GP88 expression in TCs and in ICs of muscle-invasive BCa patients; Table S2: Spearman’s bivariate correlation between GP88 staining in TCs or ICs and clinicopathological/molecular parameters in BCa patients; Table S3: Univariate Cox’s regression analysis: association of clinicopathological and molecular parameters with prognosis.

## Abbreviations

GP88 (GRN)	progranulin
BCa	bladder cancer
TCs	tumor cells
ICs	immune cells
IHC	immunohistochemistry
OS	overall survival
DSS	disease-specific survival
RFS	recurrence free survival
CD3	CD3 antigen
CD8	CD8 antigen
PD1 (PDCD1)	Programmed cell death protein 1
PD-L1 (CD274)	Programmed cell death ligand 1
CD68	CD68 antigen
CD163	CD163 antigen
CCL2	chemokine CC motif ligand 2
pT	pathological tumor stage
pN	pathological lymph node stage
n.s.	not significant
DN	double negative (for basal and luminal markers)
MUPC	micropapillary urothelial carcinoma
NOS	not otherwise specified (morphology)
PUC	plasmacytoid urothelial carcinoma

## References

[1] Sung H., Ferlay J., Siegel R.L., Laversanne M., Soerjomataram I., Jemal A., Bray F. (2021). Global cancer statistics 2020: GLOBOCAN estimates of incidence and mortality worldwide for 36 cancers in 185 countries. CA Cancer J. Clin..

[2] Sanli O., Dobruch J., Knowles M.A., Burger M., Alemozaffar M., Nielsen M.E., Lotan Y. (2017). Bladder cancer. Nat. Rev. Dis. Primers.

[3] Cheng L., Weaver A.L., Leibovich B.C., Ramnani D.M., Neumann R.M., Scherer B.G., Nehra A., Zincke H., Bostwick D.G. (2000). Predicting the survival of bladder carcinoma patients treated with radical cystectomy. Cancer.

[4] Witjes J.A., Bruins H.M., Cathomas R., Comperat E.M., Cowan N.C., Gakis G., Hernandez V., Linares Espinos E., Lorch A., Neuzillet Y. (2021). European Association of Urology Guidelines on Muscle-invasive and Metastatic Bladder Cancer: Summary of the 2020 Guidelines. Eur. Urol..

[5] Sharma P., Shen Y., Wen S., Yamada S., Jungbluth A.A., Gnjatic S., Bajorin D.F., Reuter V.E., Herr H., Old L.J. (2007). CD8 tumor-infiltrating lymphocytes are predictive of survival in muscle-invasive urothelial carcinoma. Proc. Natl. Acad. Sci. USA.

[6] Sjodahl G., Lovgren K., Lauss M., Chebil G., Patschan O., Gudjonsson S., Mansson W., Ferno M., Leandersson K., Lindgren D. (2014). Infiltration of CD3(+) and CD68(+) cells in bladder cancer is subtype specific and affects the outcome of patients with muscle-invasive tumors. Urol. Oncol..

[7] Efstathiou J.A., Mouw K.W., Gibb E.A., Liu Y., Wu C.L., Drumm M.R., da Costa J.B., du Plessis M., Wang N.Q., Davicioni E. (2019). Impact of Immune and Stromal Infiltration on Outcomes Following Bladder-Sparing Trimodality Therapy for Muscle-Invasive Bladder Cancer. Eur. Urol..

[8] Pfannstiel C., Strissel P.L., Chiappinelli K.B., Sikic D., Wach S., Wirtz R.M., Wullweber A., Taubert H., Breyer J., Otto W. (2019). The Tumor Immune Microenvironment Drives a Prognostic Relevance That Correlates with Bladder Cancer Subtypes. Cancer Immunol. Res..

[9] Bruni D., Angell H.K., Galon J. (2020). The immune contexture and Immunoscore in cancer prognosis and therapeutic efficacy. Nat. Rev. Cancer..

[10] Eckstein M., Strissel P., Strick R., Weyerer V., Wirtz R., Pfannstiel C., Wullweber A., Lange F., Erben P., Stoehr R. (2020). Cytotoxic T-cell-related gene expression signature predicts improved survival in muscle-invasive urothelial bladder cancer patients after radical cystectomy and adjuvant chemotherapy. J. Immunother. Cancer.

[11] Wang Z., Zhou Q., Zeng H., Zhang H., Liu Z., Huang Q., Xiong Y., Wang J., Chang Y., Bai Q. (2020). Tumor-infiltrating IL-17A(+) cells determine favorable prognosis and adjuvant chemotherapeutic response in muscle-invasive bladder cancer. Oncoimmunology.

[12] Bellmunt J., Powles T., Vogelzang N.J. (2017). A review on the evolution of PD-1/PD-L1 immunotherapy for bladder cancer: The future is now. Cancer Treat. Rev..

[13] Eckstein M., Wirtz R.M., Pfannstil C., Wach S., Stoehr R., Breyer J., Erlmeier F., Günes C., Nitschke K., Weichert W. (2018). A multicenter round robin test of PD-L1 expression assessment in urothelial bladder cancer by immunohistochemistry and RT-qPCR with emphasis on prognosis prediction after radical cystectomy. Oncotarget.

[14] Lopez-Beltran A., Cimadamore A., Blanca A., Massari F., Vau N., Scarpelli M., Cheng L., Montironi R. (2021). Immune Checkpoint Inhibitors for the Treatment of Bladder Cancer. Cancers.

[15] Eckstein M., Epple E., Jung R., Weigelt K., Lieb V., Sikic D., Stöhr R., Geppert C., Weyerer V., Bertz S. (2020). CCL2 Expression in Tumor Cells and Tumor-Infiltrating Immune Cells Shows Divergent Prognostic Potential for Bladder Cancer Patients Depending on Lymph Node Stage. Cancers.

[16] Buraschi S., Xu S.Q., Stefanello M., Moskalev I., Morcavallo A., Genua M., Tanimoto R., Birbe R., Peiper S.C., Gomella L.G. (2016). Suppression of progranulin expression inhibits bladder cancer growth and sensitizes cancer cells to cisplatin. Oncotarget.

[17] Wex T., Kuester D., Schönberg C., Schindele D., Treiber G., Malfertheiner P. (2011). Mucosal Progranulin expression is induced by H. pylori, but independent of Secretory Leukocyte Protease Inhibitor (SLPI) expression. BMC Gastroenterol..

[18] Bateman A., Belcourt D., Bennett H., Lazure C., Solomon S. (1990). Granulins, a novel class of peptide from leukocytes. Biochem. Biophys. Res. Commun..

[19] Okura H., Yamashita S., Ohama T., Saga A., Yamamoto-Kakuta A., Hamada Y., Sougawa N., Ohyama R., Sawa Y., Matsuyama A. (2010). HDL/apolipoprotein A-I binds to macrophage-derived progranulin and suppresses its conversion into proinflammatory granulins. J. Atheroscler. Thromb..

[20] He Z., Ismail A., Kriazhev L., Sadvakassova G., Bateman A. (2002). Progranulin (PC-cell-derived growth factor/acrogranin) regulates invasion and cell survival. Cancer Res..

[21] He Z., Bateman A. (2003). Progranulin (granulin-epithelin precursor, PC-cell-derived growth factor, acrogranin) mediates tissue repair and tumorigenesis. J. Mol. Med..

[22] Tanimoto R., Lu K.G., Xu S.Q., Buraschi S., Belfiore A., Iozzo R.V., Morrione A. (2016). Mechanisms of Progranulin Action and Regulation in Genitourinary Cancers. Front. Endocrinol..

[23] Serrero G. (2016). Potential of Theranostic Target Mining in the Development of Novel Diagnostic and Therapeutic Products in Oncology: Progranulin/GP88 as a Therapeutic and Diagnostic Target for Breast and Lung Cancers. Rinsho Byori..

[24] Serrero G., Ioffe O.B. (2003). Expression of PC-cell-derived growth factor in benign and malignant human breast epithelium. Hum. Pathol..

[25] Serrero G., Hawkins D.M., Yue B., Ioffe O., Bejarano P., Phillips J.T., Head J.F., Elliott R.L., Tkaczuk K.R., Godwin A.K. (2012). Progranulin (GP88) tumor tissue expression is associated with increased risk of recurrence in breast cancer patients diagnosed with estrogen receptor positive invasive ductal carcinoma. Breast. Cancer Res..

[26] Edelman M.J., Feliciano J., Yue B., Bejarano P., Io_e O., Reisman D., Hawkins D., Gai Q., Hicks D., Serrero G. (2014). GP88 (progranulin): A novel tissue and circulating biomarker for non-small cell lung carcinoma. Hum. Pathol..

[27] Abella V., Pino J., Scotece M.J., Lago F., Gonzalez-Gay M.A., Mera A., Gómez R., Mobasheri A., Gualillo O. (2017). Progranulin as a biomarker and potential therapeutic agent. Drug Discov. Today..

[28] Yamamoto Y., Goto N., Takemura M., Yamasuge W., Yabe K., Takami T., Miyazaki T., Takeuchi T., Shiraki M., Shimizu M. (2017). Association between increased serum GP88 (progranulin) concentrations and prognosis in patients with malignant lymphomas. Clin. Chim. Acta.

[29] Abdulrahman A., Eckstein M., Jung R., Guzman J., Weigelt K., Serrero G., Yue B., Geppert C., Stöhr R., Hartmann A. (2019). Expression of GP88 (Progranulin) Protein Is an Independent Prognostic Factor in Prostate Cancer Patients. Cancers.

[30] Koo D.H., Do I.G., Oh S., Lee Y.G., Kim K., Sohn J.H., Park S.K., Yang H.J., Jung Y.S., Park D.I. (2020). KBSMC Colorectal Cancer Team. Prognostic Value of Progranulin in Patients with Colorectal Cancer Treated with Curative Resection. Pathol. Oncol. Res..

[31] Han J.J., Yu M., Houston N., Steinberg S.M., Kohn E.C. (2011). Progranulin is a potential prognostic biomarker in advanced epithelial ovarian cancers. Gynecol. Oncol..

[32] Li G., Dong T., Yang D., Gao A., Luo J., Yang H., Wang L. (2018). Progranulin promotes lymphangiogenesis through VEGF-C and is an independent risk factor in human esophageal cancers. Hum. Pathol..

[33] Koo D.H., Park C.Y., Lee E.S., Ro J., Oh S.W. (2012). Progranulin as a prognostic biomarker for breast cancer recurrence in patients who had hormone receptor-positive tumors: A cohort study. PLoS ONE.

[34] Hu Y., Lin D.M., Cheng S.J., Liu Y.N., Feng F.Y. (2006). Influences of PC cell-derived growth factor and breast cancer resistance protein on the curative effects of platinum-based chemotherapeutic regimens for advanced non-small cell lung cancer. Zhonghua Yi Xue Za Zhi.

[35] Monami G., Gonzalez E.M., Hellman M., Gomella L.G., Baffa R., Iozzo R.V., Morrione A. (2006). Proepithelin promotes migration and invasion of 5637 bladder cancer cells through the activation of ERK1/2 and the formation of a paxillin/FAK/ERK complex. Cancer Res..

[36] Lovat F., Bitto A., Xu S.Q., Fassan M., Goldoni S., Metalli D., Wubah V., McCue P., Serrero G., Gomella L.G. (2009). Proepithelin is an autocrine growth factor for bladder cancer. Carcinogenesis.

[37] Buraschi S., Neill T., Xu S.Q., Palladino C., Belfiore A., Iozzo R.V., Morrione A. (2020). Progranulin/EphA2 axis: A novel oncogenic mechanism in bladder cancer. Matrix Biol..

[38] Selmy M.A., Ibrahim G.H., El Serafi T.I., Ghobeish A.A. (2010). Evaluation of urinary proepithelin as a potential biomarker for bladder cancer detection and prognosis in Egyptian patients. Cancer Biomark..

[39] Soukup V., Kalousová M., Capoun O., Sobotka R., Breyl Z., Pešl M., Zima T., Hanuš T. (2015). Panel of Urinary Diagnostic Markers for Non-Invasive Detection of Primary and Recurrent Urothelial Urinary Bladder Carcinoma. Urol Int..

[40] Nolte S., Zlobec I., Lugli A., Hohenberger W., Croner R., Merkel S., Hartmann A., Geppert C.I., Rau T.T. (2017). Construction and analysis of tissue microarrays in the era of digital pathology: A pilot study targeting CDX1 and CDX2 in a colon cancer cohort of 612 patients. J. Pathol. Clin. Res..

[41] Hendry S., Salgado R., Gevaert T., Russell P.A., John T., Thapa B., Christie M., van de Vijver K., Estrada M.V., Gonzalez-Ericsson P.I. (2017). Assessing tumor-infiltrating lymphocytes in solid tumors: A practical review for pathologists and proposal for a standardized method from the International Immuno-Oncology Biomarkers Working Group: Part 2: TILs in melanoma, gastrointestinal tract carcinomas, non-small cell lung carcinoma and mesothelioma, endometrial and ovarian carcinomas, squamous cell carcinoma of the head and neck, genitourinary carcinomas, and primary brain tumors. Adv. Anat. Pathol..

[42] Choi W., Porten S., Kim S., Willis D., Plimack E.R., Hoffman-Censits J., Roth B., Cheng T., Tran M., Lee I.L. (2014). Identification of distinct basal and luminal subtypes of muscle-invasive bladder cancer with different sensitivities to frontline chemotherapy. Cancer Cell..

[43] Dadhania V., Zhang M., Zhang L., Bondaruk J., Majewski T., Siefker-Radtke A., Guo C.C., Dinney C., Cogdell D.E., Zhang S. (2016). Meta-Analysis of the Luminal and Basal Subtypes of Bladder Cancer and the Identification of Signature Immunohistochemical Markers for Clinical Use. EBioMedicine.

[44] Wullweber A., Strick R., Lange F., Sikic D., Taubert H., Wach S., Wullich B., Bertz S., Weyerer V., Stoehr R. (2021). Bladder Tumor Subtype Commitment Occurs in Carcinoma In Situ Driven by Key Signaling Pathways Including ECM Remodeling. Cancer Res..

[45] Dvorak H.F. (2015). Tumors: Wounds that do not heal-redux. Cancer Immunol. Res..

[46] He Z., Ong C.H., Halper J., Bateman A. (2003). Progranulin is a mediator of the wound response. Nat. Med..

[47] Zhu J., Nathan C., Jin W., Sim D., Ashcroft G.S., Wahl S.M., Lacomis L., Erdjument-Bromage H., Tempst P., Wright C.D. (2002). Conversion of proepithelin to epithelins: Roles of SLPI and elastase in host defense and wound repair. Cell.

[48] Masson-Lecomte A., Rava M., Real F.X., Hartmann A., Allory Y., Malats N. (2014). Inflammatory biomarkers and bladder cancer prognosis: A systematic review. Eur. Urol..

[49] Nassif E.F., Thibault C., Oudard S., Galon J. (2021). Precision immunity: Immunoscore and neoadjuvant treatment in bladder cancer. Oncoimmunology.

[50] Jian J., Li G., Hettinghouse A., Liu C. (2018). Progranulin: A key player in autoimmune diseases. Cytokine.

[51] Park B., Buti L., Lee S., Matsuwaki T., Spooner E., Brinkmann M.M., Nishihara M., Ploegh H.L. (2011). Granulin is a soluble cofactor for toll-like receptor 9 signaling. Immunity.

[52] Jian J., Konopka J., Liu C. (2013). Insights into the role of progranulin in immunity, infection, and inflammation. J. Leukoc Biol..

[53] Pickford F., Marcus J., Camargo L.M., Xiao Q., Graham D., Mo J.R., Burkhardt M., Kulkarni V., Crispino J., Hering H. (2011). Progranulin is a chemoattractant for microglia and stimulates their endocytic activity. Am. J. Pathol..

[54] Ellyard J.I., Simson L., Parish C.R. (2007). Th2-mediated anti-tumour immunity: Friend or foe?. Tissue Antigens.

[55] Liu C.J., Bosch X. (2012). Progranulin: A growth factor, a novel TNFR ligand and a drug target. Pharmacol. Ther..

[56] Salomon B.L., Leclerc M., Tosello J., Ronin E., Piaggio E., Cohen J.L. (2018). Tumor Necrosis Factor α and Regulatory T Cells in Oncoimmunology. Front. Immunol..

[57] Guha R., Yue B., Dong J., Banerjee A., Serrero G. (2021). Anti-progranulin/GP88 antibody AG01 inhibits triple negative breast cancer cell proliferation and migration. Breast Cancer Res. Treat..

