# Supplementary material for: Combination of GP88 Expression in Tumor Cells and Tumor-Infiltrating Immune Cells Is an Independent Prognostic Factor for Bladder Cancer Patients
Source: Cells. 2021 Jul 15;10(7):1796. doi: 10.3390/cells10071796 (PMC8306318; doi:10.3390/cells10071796)
Supplement: Supplementary file 1 [file cells-10-01796-s001.zip › cells-1272804-supplementary.pdf]

Supplementary Materials

Combination of GP88 Expression in Tumor Cells and Tumor-Infiltrating Immune Cells is an Independent Prognostic Factor for Bladder Cancer Patients

Markus Eckstein [1,5], Verena Lieb [2], Rudolf Jung [1], Katrin Weigelt [2], Danijel Sikic [2,5], Robert Stöhr [1,5], Carol Geppert [1], Veronika Weyerer [1], Simone Bertz [1], Ginette Serrero [3,4], Arndt Hartmann [1,5], Bernd Wullich [2,5], Helge Taubert [2,5,*,†] and Sven Wach [2,5,†]

Suppl. Table S1: GP88 expression in TCs and in ICs of muscle invasive BCa patients

	Sum	Immune cells GP88 positive	Immune cells GP88 negative
Tumor cells GP88 positive (in %)	93	30 (32.3)	63 (67.7)
Tumor cells GP88 negative (in %)	103	62 (60.2)	41 (39.8)
Sum	196	92 (46.9)	104 (53.1)

Suppl. Table S2: Spearman's bivariate correlation between GP88 staining in TCs or ICs and clinicopathological/molecular parameters in BCa patients

**Parameter**		**GP88 in TCs**		**GP88 in ICs**
**Gender**	r_s	-0.036	r_s	0.053
	P	0.618	P	0.462
**Age at cystectomy**	r_s	0.040	r_s	0.019
	P	0.580	P	0.795
**Tumor grade**	r_s	0.039	r_s	- 0.171
	P	0.584	P	**0.017**
**pT**	r_s	0.192*	r_s	- 0.134
	P	**0.007**	P	0.062
**pN**	r_s	0.276**	r_s	- 0.196*
	P	**<0.001**	P	**0.006**
**Adj. chemotherapy**	r_s	0.164*	r_s	- 0.189*
	P	**0.021**	P	**0.008**
**OS**	r_s	- 0.249**	r_s	0.273**
	P	**<0.001**	P	**<0.001**
**DSS**	r_s	- 0.343**	r_s	0.483**
	P	**<0.001**	P	**<0.001**
**Survival time**	r_s	- 0.130	r_s	0.321**
	P	0.070	P	**<0.001**
**Recurrence free survival time**	r_s	- 0.141*	r_s	0.356**
	P	**0.049**	P	**<0.001**
**RFS**	r_s	- 0.384	r_s	0.495**
	P	<0.001	P	**<0.001**
**CCL2 in ICs**	r_s	- 0.181	r_s	0.349**
	P	0.020	P	**<0.001**
**CK5**	r_s	- 0.116	r_s	0.250**
	P	0.105	P	**<0.001**
**CK20**	rs	0.062		-0.189*
	P	0.386		**0.008**
**Stromal TILs**	r_s	- 0.216*	r_s	0.424**
	P	**0.002**	P	**<0.001**
**CD3**	r_s	- 0.234*	r_s	0.313**
	P	**0.001**	P	**<0.001**
**CD8**	r_s	- 0.200*	r_s	0.299**
	P	**0.005**	P	**<0.001**
**CD68**	r_s	- 0.065	r_s	0.395**
	P	0.369	P	**<0.001**

**CD163**	r_s	- 0.001	r_s	0.253**
	P	0.993	P	**<0.001**
**PD-1**	r_s	- 0.216*	r_s	0.395**
	P	**0.002**	P	**<0.001**
**PD-L1 in IC**	r_s	- 0.248	r_s	0.437**
	P	**<0.001**	P	**<0.001**
**PD-L1 in TC**	r_s	- 0.124	r_s	0.150*
	P	0.082	P	**0.036**
**GP88 in ICs/TCs**	r_s	- 0.369**	r_s	- 0.369**
	P	**<0.001**	P	**<0.001**
**molecular subtypes**	r_s	0.159*	r_s	- 0.349**
	P	**0.027**	P	**<0.001**

Significant values are marked in bold.
rs=correlation coefficient, P=P-value (2-sided), *P<0.05, **P<0.001

Suppl. Table S3: Univariate Cox's regression analysis: Association of clinicopathological and molecular parameters with prognosis

	univariate Cox's regression analysis							
	**N**	**OS**		**DSS**		**N**	**RFS**	
		RR	P	RR	P		RR	P
**Age**								
>71 years vs. <=71 years	196	1.50	**0.016**	n.s.	n.s.	195	n.s.	n.s.
**Gender**								
Female vs. male	196	1.44	**0.043**	1.54	**0.035**	195	1.48	(0.052)
**Pathol. Tumor Stage**								
pT3+4 vs pT2	196	2.08	**<0.001**	2.39	**<0.001**	195	2.30	**0.001**
**Pathol. Nodal Stage**								
pN0 (reference)	124					123		
pN1+2	57	2.37	**<0.001**	2.76	**<0.001**	57	2.76	**<0.001**
pNX	15	4.15	**<0.001**	5.56	**<0.001**	15	5.38	**<0.001**
**Chemotherapy**								
CT+ vs. CT-	196	n.s.	n.s.	n.s.	n.s.	195	n.s.	n.s.
**Molecular Subtype**								
basal (reference)	88					88		
double negative	11	1.78	(0.093)	2.04	(0.067)	11	1.96	(0.082)
luminal	75	n.s.	n.s.	n.s.	n.s.	74	n.s.	n.s.
luminal EMT-p53-like	21	n.s.	n.s.	n.s.	n.s.	21	n.s.	n.s.

Abbreviations: CT- adjuvant chemotherapy; n.s.—not significant.

Significant values are in bold
